# Evaluation of Tumor-Infiltrating Lymphocytes as Predictors of Response to Neoadjuvant Chemotherapy in Patients With Locally Advanced Breast Cancer

**DOI:** 10.7759/cureus.73133

**Published:** 2024-11-06

**Authors:** Deepak Kumar, Shivani B Paruthy, Amit Yadav, Soni Pal, Vikas Pandurangappa, Sushant Tanwar, Sajith K Mohan, Rajguru Siwach, Tulsi Appat, Prudhvi Raju TRS

**Affiliations:** 1 Department of General Surgery, Vardhman Mahavir Medical College and Safdarjung Hospital, Delhi, IND; 2 Department of Pathology, Vardhman Mahavir Medical College and Safdarjung Hospital, Delhi, IND

**Keywords:** breast cancer research, complete clinical response, locally advanced breast-cancer, neoadjuvant chemotherapy(nact), prospective observational study, recist criteria, tumor-infiltrating lymphocytes

## Abstract

Background

This study aimed to evaluate tumor-infiltrating lymphocytes (TILs) as predictors of response to neoadjuvant chemotherapy (NACT) in patients with locally advanced breast cancer (LABC).

Methods

Overall, 35 patients with LABC were included in the study. Information on demographic profile, medical history, and signs and symptoms was collected for each patient, and a complete clinical evaluation was conducted, which involved physical examination, imaging studies (mammogram/ultrasound imaging), biopsy of each patient, and a metastatic workup. Patient consent was obtained for core-needle biopsy under local anesthesia, followed by a pathologic assessment of the type of breast cancer, before NACT and after mastectomy. Patients treated with NACT were followed up for response using Response Evaluation Criteria in Solid Tumors (RECIST) version 1.1 and were scheduled for modified radical mastectomy (MRM) on completion of NACT. MRM specimens were sent for immunohistopathologic analysis for CD3 and CD5, and for grading. Subsequently, correlations between TILs and grading with NACT response and type of cancer were analyzed.

Results

Of the 35 patients, 24 were positive for CD3. A correlation was identified between NACT in LABC patients and CD3 TILs, as 68.6% of patients were CD3-positive, with 54.3% showing stromal CD3 variants and 14.3% showing intramural CD3 variants. This result indicates that CD3 TILs can be an indicator of response to NACT in LABC patients. In the sample, 48.6% of patients showed CD5 positivity, with stromal predominance. Overall, 17 patients (48.6%) had a RECIST complete response to NACT, 16 (45.7%) had a partial response, and 1 (2.9%) had progressive disease. Therefore, the study showed a significant response to NACT in LABC patients (p-value < 0.0001), and reductions in tumor size could be evaluated using RECIST criteria.

Conclusions

NACT had a significant effect on tumors, as shown by RECIST assessments in patients with LABC. TILs can be used as promising prognostic markers to evaluate and predict patients’ responses to NACT. Evaluating TILs is expensive but may be useful for the diagnosis and prediction of immunologic responses in breast cancer and other types of carcinomas following chemotherapy.

## Introduction

The current standard of care for breast cancer involves multimodal treatment, as 30-60% of cases show local progression [1]. Locally advanced breast cancer (LABC) is still a common breast cancer presentation in developing nations [2]. For many patients from locations severely afflicted by LABC, it is impractical to adhere to a treatment plan and for extensive and regular follow-up to be conducted. Neoadjuvant chemotherapy (NACT) is administered as a systemic therapy to reduce the chance of micrometastasis while enhancing local control and survival. LABC is staged more conservatively with NACT, and micrometastasis is treated via in-vivo chemo-sensitization. Most patients with breast cancer in developing nations such as India present with LABC.

Regression in tumor size is the endpoint of assessments used to measure patients’ true responses to NACT. Notably, the Response Evaluation Criteria in Solid Tumors (RECIST) [3] working group developed criteria to determine tumor responses to NACT, and these criteria have been updated to version 1.1 to include information from positron emission tomography scans and on pathologically enlarged lymph nodes. According to the RECIST criteria, an evaluation of all currently present lesions, divided into target lesions (i.e., those to be assessed) and nontarget lesions, should be integrated to determine each patient’s overall response to treatment. Altogether, the RECIST criteria (described in detail in the appendix) include four quantitative categories: complete response (CR), partial response (PR), progressive disease (PD), and stable disease (SD). The RECIST criteria is sufficient and appropriate for determining tumor-volume responses to chemotherapy in terms of both validity and inter-criteria reproducibility with the WHO criteria [4].

The role of the immune system in relation to breast-type solid tumors has been the subject of intense debate over recent years. The evaluation of infiltrating lymphocytes as novel prognostic and therapy-predicting factors should become a routinely performed analysis, with particular regard to the most aggressive breast cancers. Specifically, TILs are used to study the evolution of cancers that exhibit negative status enlargement of the axillary lymph nodes, typical of small, low-grade tumors. T-cell infiltration has been explored in detail as a potential suppressor of growth in breast carcinoma. In this context, T-cell infiltration of the tumor bed following NACT is a key immune modulator that is useful for determining treatment options for breast cancer, particularly for human epidermal growth factor receptor 2 (HER2)-positive and triple-negative subtypes [5].

Further, as an indicator of treatment response, lymphocytic infiltration into tumors is positively correlated with disease-free survival, distant disease-free survival, and overall survival, and negatively correlated with patient age. In the future, treating breast cancer with an immunotherapy approach may prove beneficial. For example, when treated with trastuzumab, patients with more stromal TILs in their tumors had a noticeably greater survival rate than those with high TILs but no trastuzumab [5].

In 2014, an international TIL working group evaluated the use of TILs in cancer assessment. The first step of the process was to identify the relevant tumor area for assessing TILs. Step 2 involved the definition of stromal TILs. Step 3 involved low-magnification microscopic observation of TILs. Step 4 determined the type of inflammatory infiltration to be observed. Finally, step 5 grouped individuals based on their stromal TIL levels: group A, with low TILs (1-10% stromal TILs); group B, with intermediate TILs (10-40% stromal TILs); and group C, with high TILs (40-90% stromal TILs) [5].

TILs may represent a novel predictor of response to NACT, as TIL levels determined by core-needle biopsy before NACT correlate with mastectomy specimens following NACT. Therefore, the primary objective of this study was to assess the role of TILs in predicting the response to NACT in patients with LABC. The secondary objective was to correlate tumor grade with TIL levels as a predictive marker of the clinicopathological outcomes of LABC.

## Materials and methods

Study design

This prospective, observational cohort study was conducted at the Department of General Surgery of Vardhaman Mahavir Medical College and Safdarjung Hospital, New Delhi. Institutional Review Board approval (IEC/VMMC/SJH/THESIS/2020-11/CC-131) was given on November 17, 2020, the study duration was 18 months (November 2020-June 2022), and the study population comprised all patients clinically diagnosed with LABC and who were scheduled for NACT and MRM. Exclusion criteria comprised patients with breast carcinoma and contraindications to NACT (i.e., due to toxicity or complications), patients who had previously undergone breast surgery, patients with recurrent breast carcinoma, and patients aged >75 years.

Sample size

Based on a previous report [6], a minimum sample size of 33 patients was required, allowing for a 10% error rate, as per the following formula:

4{zα2+zβ}{log(OR)}24{zα2+zβ}{log(OR)}2

 where OR (odds ratio) = 11.87, α = 0.5, and β = 2. To further minimize the error of the mean, a sample size of 35 patients was used in this study.

Study conduct

For all patients, a brief patient evaluation was performed, which included recording demographic profile, medical history, and signs and symptoms. After a complete clinical assessment, radiologic and hematologic tests including a mammogram, chest X-ray, electrocardiogram, echocardiogram, and ultrasonogram of the chest and abdomen were performed. Prior to NACT, patients underwent a thorough triple evaluation and metastatic workup. After patient consent was obtained, pathologic assessments were conducted by core-needle biopsy under local anesthesia to determine estrogen-receptor (ER), progesterone-receptor (PR), HER-2 neu status, and the type of breast cancer prior to NACT and mastectomy. After inviting patients to join the study, their responses to treatment were evaluated using RECIST criteria.

**Figure 1:**
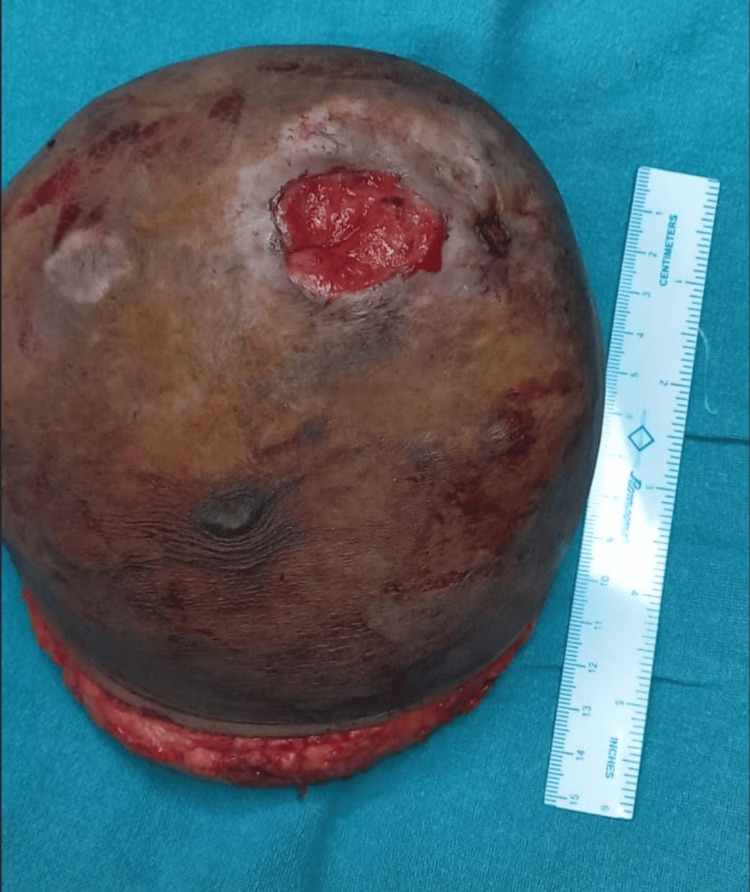
Specimen taken for histopathologic examination

Breast tissue specimens were taken for histopathologic examination (Figure 1). Then, T-lymphocyte grading was performed on formalin-fixed, paraffin-embedded tissue sections. The relevant paraffin blocks, as determined by standard diagnosis, were sliced, and slides coated with poly-L-lysine were used for sectioning. The sections were washed with alcohol of decreasing concentrations, followed by water. To prevent endogenous peroxidase activity in immunohistochemical staining and to reduce false-positive results, 3% hydrogen peroxide was added, and the slides were coated with G175-405, DO7 murine monoclonal antibodies. The slides were placed in a moist chamber and kept in a refrigerator overnight to maintain the temperature at 4°C. A chromogen detector was added after the slides had been washed with tris(hydroxymethyl)aminomethane (tris) buffer.

After the slides had been properly stained by the chromogen detector, they were washed again with distilled water and tris buffer. Hematoxylin and eosin were added as a counterstain for 10-15 seconds, and the slides were washed again with tris buffer. The tissue was dehydrated as the alcohol concentration increased. Coverslips were positioned after mounting the slides on distrene, plasticizer, and xylene. The T-cell type (CD3 or CD5) in breast carcinoma and the correlation between TIL-positivity and tumor response to NACT were evaluated.

**Figure 2:**
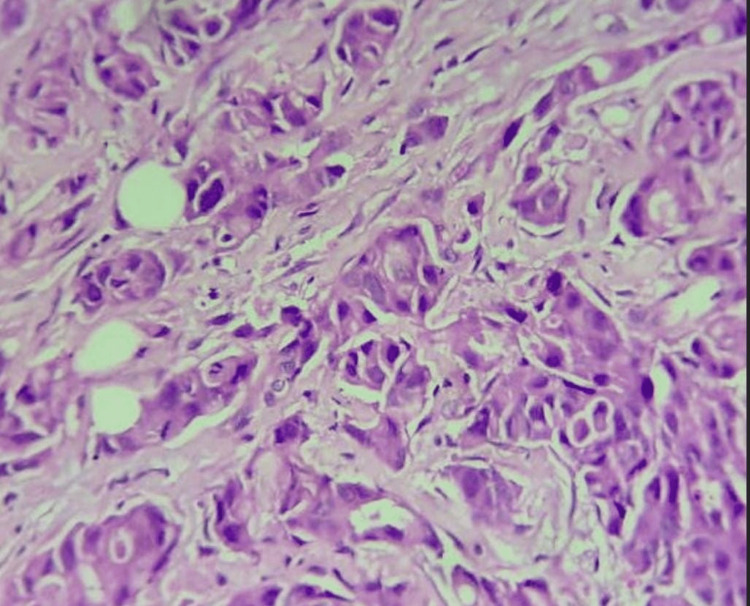
Photomicrograph shows malignant epithelial cells arranged in tubules, clusters, and in sheets showing moderate pleomorphism - invasive breast carcinoma, NST (Modified Bloom Richardson score-grade 2) (Hematoxylin and eosin stain, 400×) NST: No special type

Figure 2 shows the specimen of breast tissue stained with hematoxylin and eosin.

Statistical analyses

Categoric variables are presented as numbers and percentages, and continuous variables are presented as mean ± SD and median. Diagnostic tests were used to calculate the sensitivity, specificity, positive predictive value, and negative predictive value. Inter-rater kappa agreement was used to identify the strength of agreement between the predicted response and the actual response. A p-value <0.05 was considered statistically significant. The data were entered into an Excel spreadsheet (Microsoft Corporation, Redmond, WA, USA), and the analyses were carried out using SPSS version 21.0 (IBM Corp., Armonk, NY, USA).

## Results

A total of 35 female, married participants were included in the study. Age ranged from 32 to 70 years, with a mean ± SD of 48.8 ± 10.7 years. The mean ± SD age at menarche was 13.2 ± 1.0 (range 12-16) years. Most patients (20/35; 57.1%) had a breast lump on the left side. Fixity of the lump was not seen in two patients (5.7%), but fixity was seen in the skin in 28 patients (80%), in the pectoralis major in four patients (11.4%), and in both the skin and pectoralis major in one patient (2.9%).

The most common site for breast lumps was the upper inner or outer quadrant of the breast (11/35; 31.4%). Ten patients (28.6%) had normal nipples, retraction was seen in 24 patients (68.6%), and retraction and discharge were present in one patient (2.9%). Mobile supraclavicular nodes were seen in only one patient (2.9%). All patients had invasive breast carcinoma (IBC), no specific type (NST).

ER status was negative in 17 patients (48.6%) and positive in 18 (51.4%), indicating no clear majority of ER positivity in the sample. PR status was negative in 22 patients (62.9%) and positive in 13 (37.1%), indicating a clear predominance of PR negativity in the sample. HER2 status was negative in 22 patients (62.9%) and positive in 13 (37.1%).

The mean ± SD modified Bloom Richardson (MBR) score was 8.0 ± 0.5 (range 7-9). Overall, four patients had an MBR score of 7, with grade 2 moderately differentiated tumors; 31 patients had poorly differentiated tumors (grade 3).

The surrogate molecular class of HER2-enriched breast cancer was seen in 10 patients (28.6%). Luminal A breast cancer was evident in two patients (5.7%), luminal B in 15 (42.9%), and triple-negative breast cancer in eight patients (22.9%). Abdominal and thoracic contrast-enhanced computed tomography was conducted for all patients to rule out metastasis. The prevalence of metastasis was 25.7% (9/35 patients).

**Figure 3:**
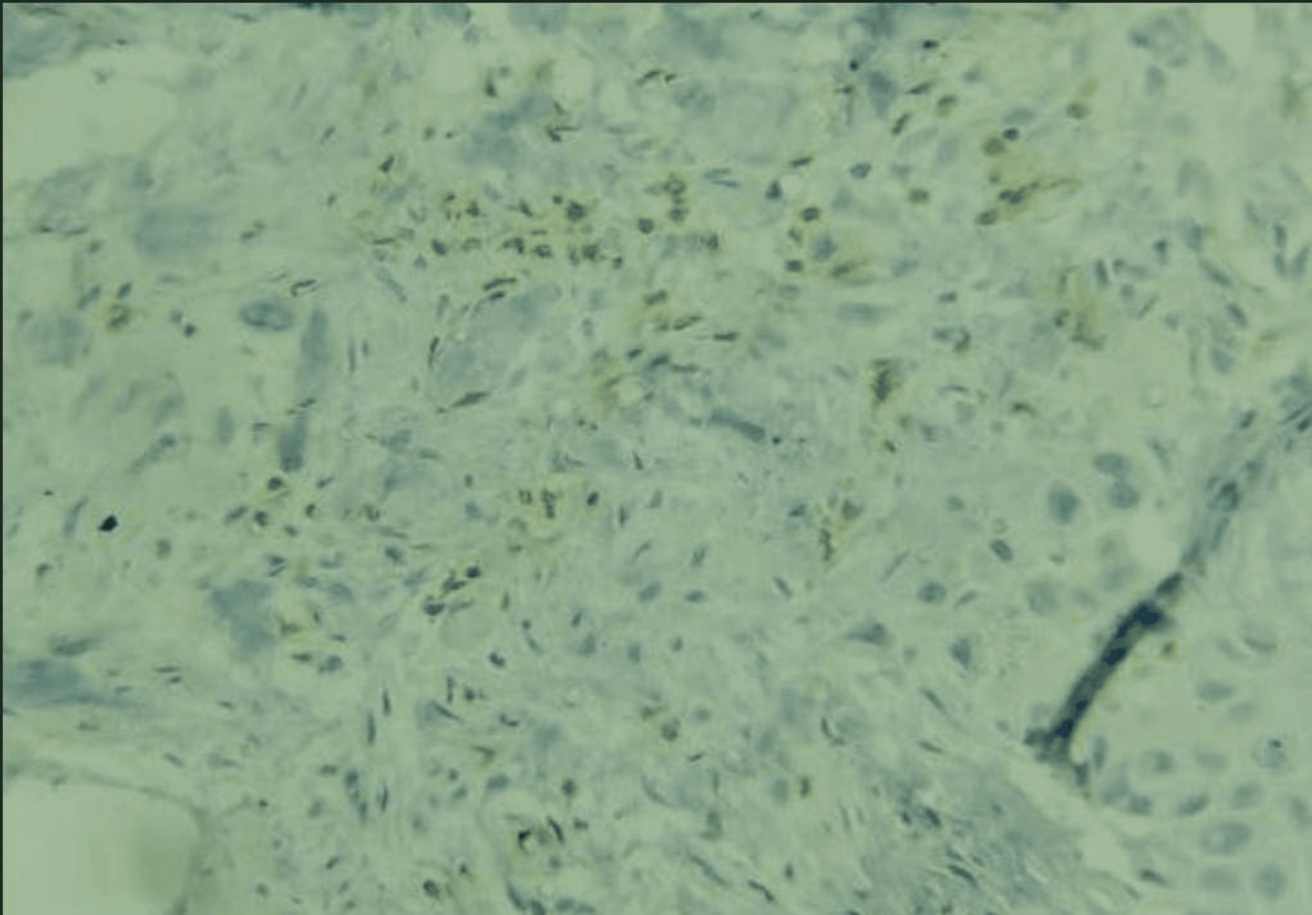
IHC showing intratumoral lymphocytes stained by CD3 IHC (IHC, 100×) IHC: immunohistochemistry

**Figure 4:**
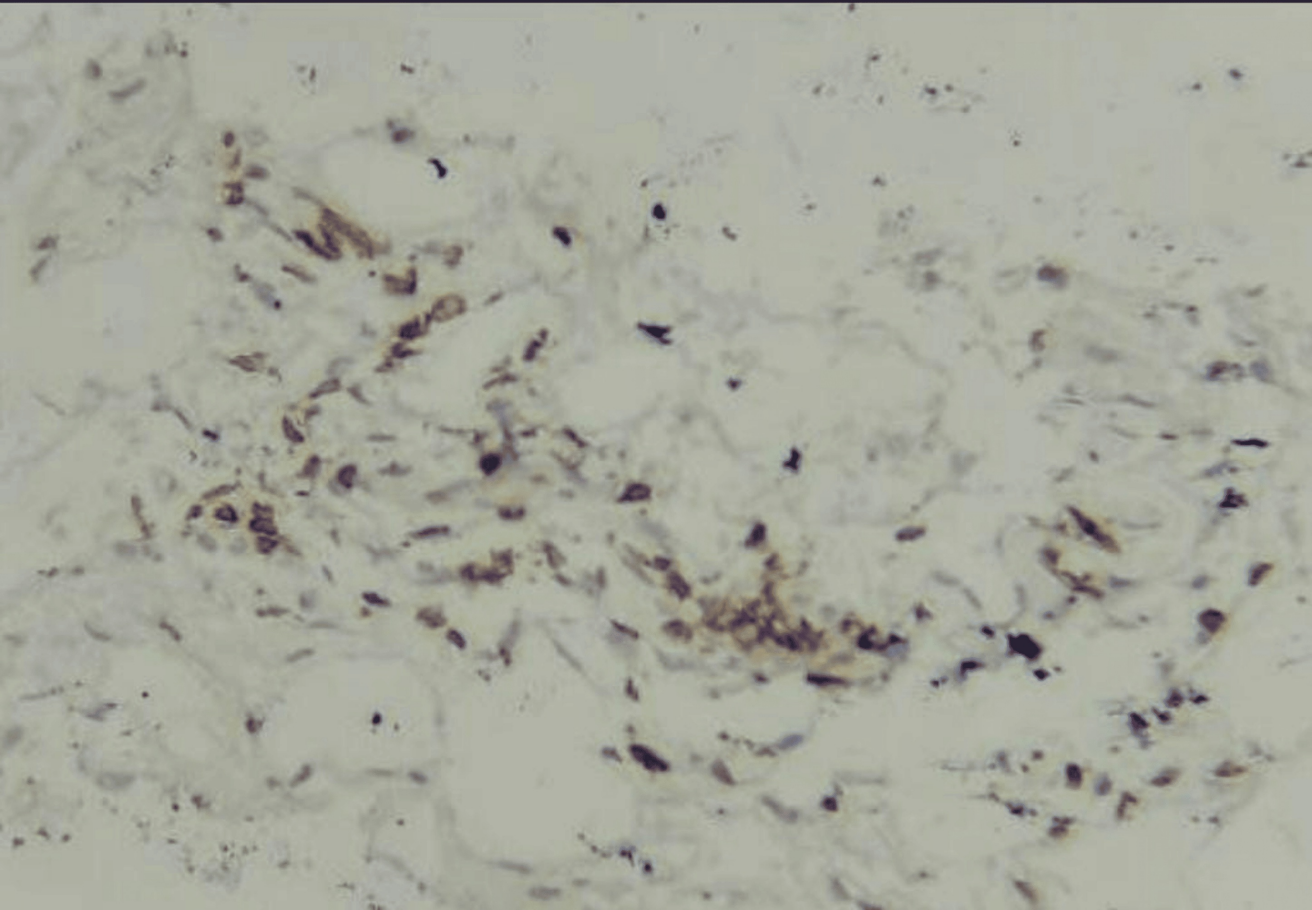
IHC shows stromal lymphocytes stained by CD3 IHC (IHC, 400×) IHC: immunohistochemistry

Of the 35 patients, three (8.7%) were at clinical stage 3a, 24 (68.6%) were at stage 3b, and eight (22.9%) were at stage 4. Overall, 24/35 patients (68.6%) were CD3-positive, and 11 (31.4%) were CD3-negative. Five patients (14.3%) had intratumoral or intraepithelial infiltration of CD3 cells (Figure 3), whereas 19 patients (54.3%) had stromal infiltration of CD3 cells (Figure 4).

**Figure 5:**
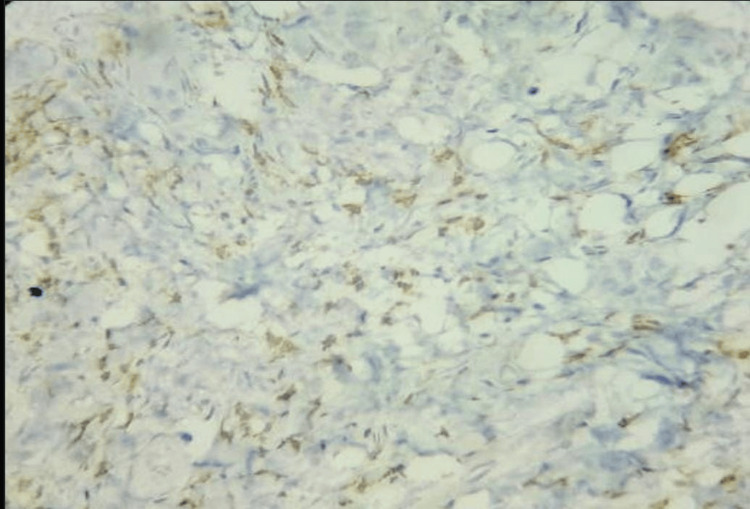
IHC shows intratumoral lymphocytes stained by CD5 IHC (IHC, 100×) IHC: immunohistochemistry

**Figure 6:**
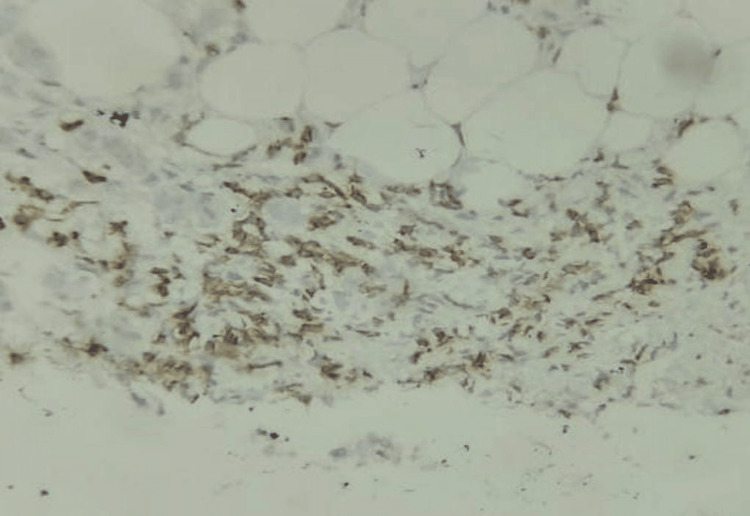
IHC shows stromal lymphocytes stained by CD5 IHC (IHC, 400×) IHC: immunohistochemistry

Altogether, 17 patients (48.6%) were CD5-positive, 14 (40.0%) were CD5-negative, and CD5 characteristics could not be assessed in 4 patients (11.4%). Three patients (8.6%) had intratumoral or intraepithelial infiltration of CD5 cells (Figure 5), whereas 13 patients (37.1%) had stromal infiltration of CD5 cells (Figure 6); in one CD5-positive patient (2.9%), a predominance of intratumoral/intraepithelial or stromal infiltration of CD5 cells could not be determined.

Table 1 shows the statistically significant reductions (p<0.0001) in breast lump size from before to after NACT in the transverse, craniocaudal, and anteroposterior planes.

**Table 1:** Comparison of breast lump size before and after neoadjuvant chemotherapy SD: standard deviation; SEM: standard error of the mean *Statistical significance (paired t-test) for each plane.

		Mean	N	Std. Deviation	Std. Error Mean	P-value*
Before chemotherapy	Transverse plane	61.84	35	15.22	3.04	<0.0001
After chemotherapy		34.44	35	12.86	2.57	
Before chemotherapy	Craniocaudal plane	52.92	35	13.18	2.63	<0.0001
After chemotherapy		25.44	35	10.58	2.11	
Before chemotherapy	Anteroposterior plane	39.36	35	10.31	2.06	<0.0001
After chemotherapy		20.48	35	9.40	1.88	

According to RECIST criteria, a CR was seen in 17 patients (48.6%), a partial response in 16 (45.7%), and PD in one patient (2.9%) following NACT. No patients had SD, and one patient (2.9%) was lost to follow-up.

No significant relationship was observed between the presence of CD3 cells and RECIST treatment response (Tables 2-3).

**Table 2:** Relationship between RECIST response and CD3 cells considering stromal and intratumoral TILs together IT: intatumoral predominance; RECIST: Response Evaluation Criteria in Solid Tumors; S: stromal predominance

		Response Based on RECIST Criteria			
	Unit	Complete response	Partial response	Progressive disease	Total
CD3 Negative	n (%)	7 (70.0)	3 (30.0)	0 (0.0)	10 (100.0)
CD3 Positive IT	n (%)	3 (60.0)	2 (40.0)	0 (0.0)	5 (100.0)
CD3 Positive S	n (%)	7 (36.8)	11 (57.9)	1 (5.3)	19 (100.0)
Total	n (%)	17 (50.0)	16 (47.1)	1 (2.9)	34 (100.0)
P-value					0.479

**Table 3:** Relationship between RECIST response and CD3 cells RECIST: Response Evaluation Criteria in Solid Tumors; TIL: tumor-infiltrating lymphocytes The chi-square test was done with a p-value<0.05 being significant.

		Response Based on RECIST Criteria			
	Unit	Complete response	Partial response	Progressive disease	Total
CD3 Negative	n (%)	7 (70.0)	3 (30.0)	0 (0.0)	10 (100.0)
CD3 Positive	n (%)	10 (41.7)	13 (54.2)	1 (4.2)	24 (100.0)
Total	n (%)	17 (50.0)	16 (47.1)	1 (2.9)	34 (100.0)
P-value					0.297

No significant relationship was observed between the presence of CD3 cells and treatment response based on RECIST criteria according to a Chi-square test (p > 0.05). When using the combined values for stromal and intratumoral CD3 cells, the number of CD3-positive subjects was 24, whereas 10 subjects were found to be negative for CD3.

No significant relationship was observed between the presence of CD5 cells and RECIST treatment response (Table 4).

**Table 4:** Relationship between RECIST response and CD5 cells considering stromal and intratumoral TILs together IT: intratumoral; NA: reporting on tumor-infiltrating lymphocytes not available; S: stromal CD5 cells The chi-square test proved insignificant with a p-value of 0.620.

		Response Based on RECIST Criteria			
	Unit	Complete response	Partial response	Progressive disease	Total
CD5 Negative	n (%)	9 (69.2)	4 (30.8)	0 (0.0)	13 (100.0)
CD5 Positive (indeterminate S/IT)	n (%)	0 (0.0)	1 (100.0)	0 (0.0)	1 (100.0)
CD5 Positive IT	n (%)	2 (66.7)	1 (33.3)	0 (0.0)	3 (100.0)
CD5 Positive S	n (%)	4 (30.8)	8 (61.5)	1 (7.7)	13 (100.0)
NA	n (%)	2 (50.0)	2 (50.0)	0 (0.0)	4 (100.0)
Total	n (%)	17 (50.0)	16 (47.1)	1(2.9)	34 (100.0)
P-value					0.620

## Discussion

Over the past few decades, there has been a paradigm shift in the treatment of breast cancer. More women are being diagnosed with breast cancer, as both breast cancer awareness and life expectancy increase. Clearly, identifying and treating breast cancer is difficult due to inaccurate test results, the need for expensive equipment that may be inaccessible to many low-income populations, the threat of recurrence, and concerns about disfigurement in the minds of doctors and patients. Pathology and surgical competence are beneficial for patients. Indeed, breast cancer treatment relies heavily on modern histopathology. Using RECIST criteria, it is possible to compare responses to chemotherapy with markers for TILs in patients with LABC.

In this study, treatment response and TILs were examined in 35 patients with breast cancer. The mean(±SD) age of the study population was 48.8 ± 10.7 years, and the mean age at presentation for breast cancer in the Indian population is approximately 48-52 years [7,8]. These mean ages demonstrate that most patients diagnosed with LABC are not young. Il’yasova et al. reported that the incidence of LABC was substantially higher in women aged >50 versus <50 years [9]. Our study included women aged 20-84 years; further, Invasive breast cancer, NST was the most common histopathologic pattern, as observed in a study conducted by Naveed et al. [10].

In this study, we identified patients with luminal A, luminal B, Her 2 enriched and basal-like subtypes. Since only 18/35 patients (51.4%) were ER-positive, there was no significant majority of ER-positivity versus ER-negativity in the sample. Conversely, 22/35 patients (62.9%) were PR-negative, and most patients (22/35; 62.9%) were HER2-negative. In a study of 485 women with breast cancer by Colleoni et al., the ER/PR-negative group had 12.0 times (95% confidence interval: 4.93, 29.28) higher chance of achieving a pathologic CR (pCR) than patients in the ER/PR-positive or ER/PR-low group [11].

Most patients with LABC had an MBR score of grade 3 (i.e., poorly differentiated tumors). According to research by Vasudevan et al., histologic evaluation of the tumor bed is the gold standard for determining tumor response to chemotherapy, which is most effective in young patients with small tumors and low tumor grades [12].

In 2020, an International Expert Panel on the Use of Neoadjuvant (Primary) Systemic Treatment of Operable Breast Cancer advocated for a revision to the decision-making process for choosing neoadjuvant systemic medicines for patients with early breast cancer [13]. In our study, 24/35 patients were CD3-positive, and stromal infiltration was more common than intratumoral infiltration in terms of CD3 lymphocyte density. We identified a relationship between the use of NACT and CD3 TIL count in patients with LABC; indeed, 68.6% of participants were CD3-positive, with 54.3% of these having stromal predominance of CD3 cells and 14.3% having an intratumoral predominance. This finding suggests that CD3 TILs may be a sign of NACT response in patients with LABC.

According to a study by Koletsa et al. [13] in 1,011 patients, high individual lymphocytic subsets and TIL density were significantly (p<0.001) related to higher tumor grade, greater proliferation, and HER2-positive and triple-negative tumors. Further, higher TIL density (10% increments) was associated with high levels of tumor markers (CD3, CD4, CD5, CD8) and improved prognosis. However, the presence of stromal versus intratumoral lymphocytic subsets lost their prognostic relevance when TIL density was considered, with higher TIL density associated with a 15% lower risk of relapse. Higher overall rates of CD3-positive and CD8-positive TILs were associated with 35% and 28% reduced risks of relapse, respectively, independent of TIL density [14].

In our study, 17/35 patients (48.6%) were CD5-positive, 14 (40%) were CD5-negative, and in four patients, CD5 status could not be determined. Moreover, 13 (37.1%) of the 17 (48.6%) CD5-positive cases showed a stromal predominance of CD5 cells and 3 (8.6%) showed an intratumoral predominance of CD5 cells, and in one patient the stromal or intratumoral predominance could not be determined. These results may be due to stromal-predominant CD5 cells being more common than intratumoral-predominant CD5 cells. However, there is no evidence to support a robust correlation between CD5 cells and NACT response in patients with breast cancer. CD5-positivity correlates with tumor responses in blood malignancies, but this association does not appear to be as strong regarding NACT for breast cancer. TILs (i.e., CD3, CD5, CD4, PD-1) are not predictive markers in breast cancer, according to the few studies that have assessed the prognostic effects of TIL subsets in patients with breast cancer [14].

A previous study by He et al. demonstrated a negligible connection between CD5 positivity and treatment response in patients with IDC [15]. According to Shousha et al., the CD5 molecule (TI, Leu 1) is a 67 kDa transmembrane glycoprotein that has several characteristics similar to many growth factor receptors, such as high cysteine content in the extracellular domain, and a sizable cytoplasmic region with the potential for tyrosine phosphorylation. Almost all T-cells and a few B lymphocytes typically contain CD5 [16].

In our study, 17/35 patients (48.6%) had a full response to NACT. However, one patient (2.9%) had PD, and 16 patients (45.7%) had only a partial response. Thus, the treatment response to NACT can be observed and evaluated using RECIST criteria. Most patients displayed disease downstaging, which can be assessed using the RECIST criteria, and the study demonstrated significant responses to NACT in patients with LABC (p< 0.0001 for CR, partial response, etc.).

In research by Kamal et al., postoperative histopathology results showed that 60/81 lesions responded to treatment (Miller-Payne grades 3, 4, and 5). Conversely, using a combined response-evaluation approach (considering the 60 evaluable patients), 57/60 patients (95%) were responders, while using RECIST 1.1 criteria alone, only 46/60 patients (76.7%) were classified as responders [17]. In our investigation, there were no significant relationships between RECIST treatment responses and TIL markers (i.e., CD3 and CD5). However, TILs may be a promising prognostic factor for predicting responses to NACT in patients with LABC. Indeed, a study evaluated TIL expression levels before and after NAC in patients who did not achieve a pCR and found that patients with high TIL expression in their residual tumors had considerably better prognoses than patients with low TIL expression. TILs can be used to predict responses to NACT in patients with LABC and provide information on tumor histopathologic responses. There were no discernible differences in TILs in patients aged 27-77 years in the study by Kamal et al., which re-evaluated 51 specimens from 2011-2015 (mean patient age 49.2 years). Additionally, in patients receiving NACT, there was no connection between treatment response and histopathologic grade or histologic type of breast cancer [17].

Limitations and strengths

Our study has some limitations. Firstly, the small sample size (N=35) may limit the reproducibility in larger groups. Secondly, the patients were followed for a short duration, and postoperative responses, TIL levels in residual tumors, and other TIL markers require evaluation in studies with longer follow-ups. Thirdly, our study had an observational design and randomized control trials are needed to establish the accuracy of our results. Limited research has been done in this field, and further larger-scale studies should now be conducted.

Nonetheless, ours is the first observational study investigating the use of TILs as a prognostic marker of RECIST responses to NACT in patients with LABC. This work forms a useful basis for future studies as ongoing research is focused on understanding the mechanisms by which TILs mediate their effects and the potential integration of TIL evaluation into routine clinical practice to personalize treatment strategies.

## Conclusions

Overall, 35 female patients with breast carcinoma were included in this study and underwent NACT followed by mastectomy. All the patients were diagnosed with LABC and underwent NACT, MRM, and examinations to identify TILs. NACT had a significantly favorable effect on the tumors, as evaluated using RECIST criteria (p<0.0001 for CR, PR, etc.) which proves that TILs may be a promising prognostic marker for evaluating and predicting responses to NACT, but the correlation between TILs (CD3, CD5) and RECIST treatment response was not statistically significant. Although analyzing TILs is costly, TILs not only serve as a prognostic indicator, suggesting better survival rates, but also as a predictive marker for response to NACT in breast cancer and other carcinomas.

## References

[1] Rustogi A, Budrukkar A, Dinshaw K, Jalali R (2005). Management of locally advanced breast cancer: Evolution and current practice. J Cancer Res Ther.

[2] Agarwal G, Sonthineni C, Mayilvaganan S, Mishra A, Lal P, Agrawal V (2018). Surgical outcomes of primary versus post-neoadjuvant chemotherapy breast conservation surgery: A comparative study from a developing country. World J Surg.

[3] Therasse P, Arbuck SG, Eisenhauer EA (2000). New guidelines to evaluate the response to treatment in solid tumors. European Organization for Research and Treatment of Cancer, National Cancer Institute of the United States, National Cancer Institute of Canada. J Natl Cancer Inst.

[4] Eisenhauer EA, Therasse P, Bogaerts J (2009). New response evaluation criteria in solid tumours: Revised RECIST guideline (version 1.1). Eur J Cancer.

[5] Zgura A, Galesa L, Bratila E, Anghel R (2018). Relationship between tumor infiltrating lymphocytes and progression in breast cancer. Maedica (Bucur).

[6] García-Martínez E, Gil GL, Benito AC (2014). Tumor-infiltrating immune cell profiles and their change after neoadjuvant chemotherapy predict response and prognosis of breast cancer. Breast Cancer Res.

[7] Saxena S, Rekhi B, Bansal A, Bagga A, Chintamani Chintamani, Murthy NS (2005). Clinico-morphological patterns of breast cancer including family history in a New Delhi hospital, India - A cross-sectional study. World J Surg Oncol.

[8] Chin SN, Green CM, Gordon-Strachan GM, Wharfe GH (2014). Locally advanced breast cancer in Jamaica: Prevalence, disease characteristics and response to preoperative therapy. Asian Pac J Cancer Prev.

[9] Il'yasova D, Siamakpour-Reihani S, Akushevich I, Akushevich L, Spector N, Schildkraut J (2011). What can we learn from the age- and race/ethnicity- specific rates of inflammatory breast carcinoma?. Breast Cancer Res Treat.

[10] Naveed S, Quari H, Panjawani G, Shah A, Panday BB (2016). Evaluation of clinicopathological Findings on 255 cases of Inoperable Locally Advanced Breast Carcinoma: A Tertiary Care Experience. Gulf J Oncol.

[11] Colleoni M, Viale G, Zahrieh D (2008). Expression of ER, PgR, HER1, HER2, and response: A study of preoperative chemotherapy. Ann Oncol.

[12] Vasudevan D, Jayalakshmy PS, Kumar S, Mathew S (2015). Assessment of pathological response of breast carcinoma in modified radical mastectomy specimens after neoadjuvant chemotherapy. Int J Breast Cancer.

[13] Koletsa T, Kotoula V, Koliou GA (2020). Prognostic impact of stromal and intratumoral CD3, CD8 and FOXP3 in adjuvantly treated breast cancer: Do they add information over stromal tumor-infiltrating lymphocyte density?. Cancer Immunol Immunother.

[14] Mao Y, Qu Q, Chen X, Huang O, Wu J, Shen K (2016). The prognostic value of tumor-infiltrating lymphocytes in breast cancer: A systematic review and meta-analysis. PLoS ONE.

[15] He Q, Xue S, Wa Q (2021). Mining immune-related genes with prognostic value in the tumor microenvironment of breast invasive ductal carcinoma. Medicine.

[16] Shousha S, Costello C, Luqmani YA, Sinnett HD (1998). CD5 positive breast carcinoma in a patient with untreated chronic lymphocytic leukaemia: Molecular studies of chromosome 13q. J Clin Pathol.

[17] Kamal RM, Saad SM, Moustafa AFI (2020). Predicting response to neo-adjuvantchemotherapy and assessment of residual disease in breast cancer using contrast-enhanced spectral mammography: A combined qualitative and quantitative approach. Egypt J Radiol Nucl Med.

